# Tau and the fractionated default mode network in atypical Alzheimer’s disease

**DOI:** 10.1093/braincomms/fcac055

**Published:** 2022-03-09

**Authors:** Deepti Putcha, Ryan Eckbo, Yuta Katsumi, Bradford C. Dickerson, Alexandra Touroutoglou, Jessica A. Collins

**Affiliations:** 1 Frontotemporal Disorders Unit, Massachusetts General Hospital and Harvard Medical School, Boston, MA, USA; 2 Department of Psychiatry, Massachusetts General Hospital and Harvard Medical School, Boston, MA, USA; 3 Alzheimer’s Disease Research Center, Massachusetts General Hospital and Harvard Medical School, Boston, MA, USA; 4 Athinoula A. Martinos Center for Biomedical Imaging, Massachusetts General Hospital and Harvard Medical School, Boston, MA, USA

**Keywords:** resting-state fMRI, functional connectivity, posterior cortical atrophy, logopenic variant primary progressive aphasia

## Abstract

Alzheimer’s disease-related atrophy in the posterior cingulate cortex, a key node of the default mode network, is present in the early stages of disease progression across clinical phenotypic variants of the disease. In the typical amnestic variant, posterior cingulate cortex neuropathology has been linked with disrupted connectivity of the posterior default mode network, but it remains unclear if this relationship is observed across atypical variants of Alzheimer’s disease. In the present study, we first sought to determine if tau pathology is consistently present in the posterior cingulate cortex and other posterior nodes of the default mode network across the atypical Alzheimer’s disease syndromic spectrum. Second, we examined functional connectivity disruptions within the default mode network and sought to determine if tau pathology is related to functional disconnection within this network. We studied a sample of 25 amyloid-positive atypical Alzheimer’s disease participants examined with high-resolution MRI, tau (^18^F-AV-1451) PET, and resting-state functional MRI. In these patients, high levels of tau pathology in the posteromedial cortex and hypoconnectivity between temporal and parietal nodes of the default mode network were observed relative to healthy older controls. Furthermore, higher tau signal and reduced grey matter density in the posterior cingulate cortex and angular gyrus were associated with reduced parietal functional connectivity across individual patients, related to poorer cognitive scores. Our findings converge with what has been reported in amnestic Alzheimer’s disease, and together these observations offer a unifying mechanistic feature that relates posterior cingulate cortex tau deposition to aberrant default mode network connectivity across heterogeneous clinical phenotypes of Alzheimer’s disease.

## Teaser

Putcha *et al*. report high levels of tau pathology in the posteromedial and lateral temporal cortex associated with reduced functional connectivity within the default mode network across atypical Alzheimer’s disease syndromes. These observations offer a unifying mechanistic feature relating tau deposition to aberrant connectivity across the Alzheimer’s disease spectrum.

## Graphical Abstract

**Graphical Abstract:**
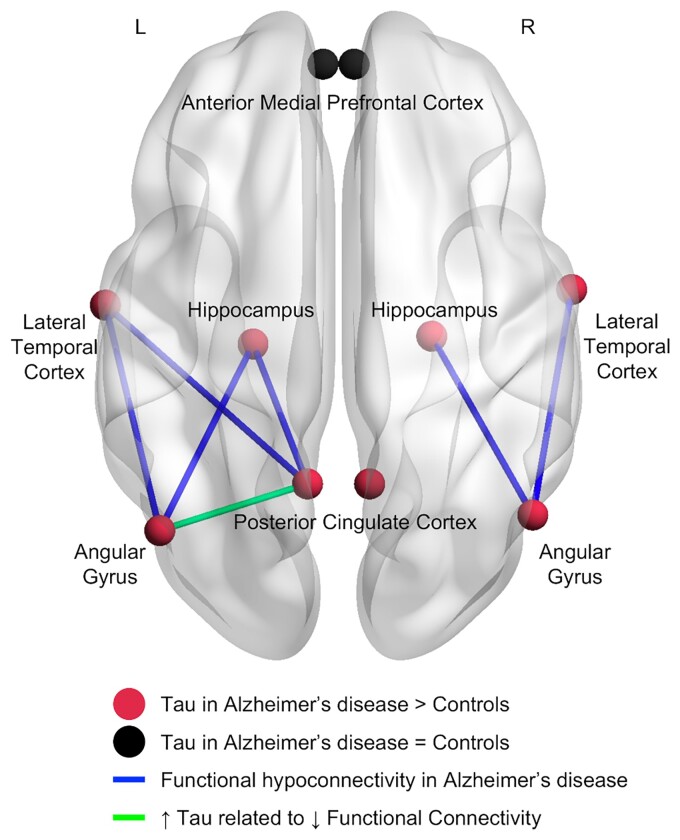

## Introduction

Patients with symptoms arising from Alzheimer’s disease have biomarker evidence of amyloid plaques, tau neurofibrillary tangles and neurodegeneration, including atrophy and network dysfunction. As research employing these biomarkers matures, relationships between them are beginning to be understood. For example, the posterior cingulate cortex (PCC), a key node of the default mode network (DMN), is a centrally affected node in Alzheimer’s disease whether measured using structural, functional or metabolic imaging.^1–6^ Several studies have reported the loss of connectivity between the PCC and other temporal, parietal and prefrontal nodes of the DMN in symptomatic Alzheimer’s disease patients.^7–10^ Although the direction of causality is not yet clear, amyloid accumulation in the posteromedial cortices including the PCC is associated with hypoconnectivity within the DMN in prodromal amnestic Alzheimer’s disease.^11,12^ However, despite the progressive loss of DMN connectivity observed over the course of Alzheimer’s disease decline, the amyloid burden in these regions remains stable over the conversion period from mild cognitive impairment to Alzheimer’s disease dementia,^13^ calling into question the utility of amyloid spread as a metric in understanding the development of symptoms in Alzheimer’s disease. Additionally, there is a disconnect between widespread amyloid retention patterns and patterns of atrophy and glucose hypometabolism which give rise to the clinical and anatomical heterogeneity in Alzheimer’s disease.^14–16^ In contrast, the patterns of tau accumulation are consistent with the heterogeneous clinical symptomatology and topology of neurodegeneration seen across the Alzheimer’s disease phenotypic spectrum.^17–19^ Recent multimodal imaging studies have reported that functional connectivity changes within the DMN and other large-scale networks in Alzheimer’s disease co-localize with patterns of neurofibrillary tau accumulation such that tau covariance patterns reflect intrinsic functional connectivity maps in young adults.^20^ Further evidence linking tau and functional connectivity is that regions that are strongly functionally connected to high tau regions will themselves carry high levels of hyperphosphorylated tau aggregates^21^ in support of the cascading network failure hypothesis.^22^ Although the nature of the relationship between tau protein accumulation and functional connectivity changes in Alzheimer’s disease is not entirely clear, some recent work suggests that increased tau PET uptake is related to hypoconnectivity within the DMN in early stages of typical amnestic Alzheimer’s disease^23^ and patients diagnosed with dementia with Lewy bodies who have co-occurring Alzheimer’s disease pathology.^24^ Despite these advances in our understanding of the relationships between Alzheimer’s disease biomarkers, much of this work has focused on patients with typical amnestic symptoms, which is only one part of the spectrum of the illness. To the best of our knowledge, no study to date has examined how tau accumulation within the PCC or other core nodes of the DMN affects within-network connectivity across atypical phenotypes of Alzheimer’s disease.

While patients with cognitive impairment arising from Alzheimer’s disease most commonly exhibit progressive amnesia evolving into multi-domain dementia,^25^ less common atypical clinical presentations of Alzheimer’s disease include progressive visual and/or spatial dysfunction (posterior cortical atrophy; PCA),^26^ progressive aphasia (usually the logopenic variant of primary progressive aphasia; lvPPA)^27^ and early-onset progressive executive, or less commonly behavioural, dysfunction.^28,29^ Although the medial temporal lobes (MTLs) are thought to be the site of earliest tangle pathology in typical Alzheimer’s disease, tau-related neuropathological changes are also detectable in other limbic and heteromodal cortical regions at relatively early stages of disease progression.^30,31^ Indeed, atypical clinical phenotypes are associated with a relative sparing of MTL regions and prominent Alzheimer’s disease tau-related neuropathological changes in isocortical regions, including lateral temporal and parietal regions.^32^ These posterior cortical regions have also been highlighted as sites of neurodegeneration and tau accumulation by neuroimaging studies in atypical phenotypes of Alzheimer’s disease. Specifically, the PCC is one of the most prominent regions of atrophy in early-onset Alzheimer’s disease cases who often present with atypical clinical phenotypes.^33–36^ Recent evidence has also suggested that the PCC has been established as a region of significant tau accumulation over time in PCA^17^ and lvPPA.^14,37^ Because the PCC and neighbouring precuneus are hub regions with a high level of connectivity to the rest of the DMN,^38,39^ tau pathology and neurodegeneration localized here likely causes disruptions to the functional connectivity of the DMN across the phenotypic spectrum of Alzheimer’s disease. Using a graph-theoretical approach, a recent study showed that regions with high levels of tau were more likely to have low clustering coefficients, suggesting vulnerability to local network failures in atypical Alzheimer’s disease.^40^

The goal of the current study is to determine if tau pathology within the PCC and other posterior cortical nodes of the DMN, regions of early neuropathology and neurodegeneration across clinical phenotypes of symptomatic Alzheimer’s disease, impacts functional connectivity within the DMN in a group of atypical Alzheimer’s disease patients. Though the primary drivers of the earliest symptoms in these atypical phenotypes of Alzheimer’s disease are observed in other domain-specific networks,^41^ we hypothesized that the DMN is also a critically affected network across the atypical Alzheimer’s disease phenotypic spectrum. Building on prior work in typical older-onset amnestic Alzheimer’s disease demonstrating the deleterious effect of Alzheimer’s disease neuropathology in the PCC on functional connectivity of the DMN,^11,12^ we hypothesized that increased tau in the PCC would be related to reduced posterior DMN connectivity across our group of atypical Alzheimer’s disease presentations. We also examined the grey matter (GM) density of the posterior nodes of the DMN to determine if tau pathology impacts functional connectivity over and above effects of volume loss in the same regions. Examining the relationship between tau pathology and functional network connectivity within the DMN may help explain how early sites of tau deposition bring about aberrant network connectivity in atypical Alzheimer’s disease, likely contributing to the cognitive dysfunction observed across the clinical spectrum of Alzheimer’s disease. As biomarkers of the underlying pathobiology of Alzheimer’s disease continue to mature, we seek to understand the relationships between them across the full phenotypic spectrum of cognitive impairment in Alzheimer’s disease, recognizing the continuous nature of this phenotypic spectrum rather than the categorical classification of these syndromes.^42^

## Materials and methods

### Participants

Twenty-five individuals with amyloid-beta positive (Aβ+) status were recruited from the Massachusetts General Hospital (MGH) Frontotemporal Disorders Unit, including the PPA and PCA programmes.^43^ See Table 1 for demographic and clinical data. All patients received a standard clinical evaluation comprising a comprehensive neurological and psychiatric history and examination and structured informant interviews. Clinical formulation was performed through consensus discussions by our multidisciplinary team, with each patient being classified based on all available clinical information as having mild cognitive impairment or dementia (global cognitive status), followed by each patient’s cognitive behavioural syndrome being diagnosed.^44^ Thirteen Aβ+ patients met diagnostic criteria for PCA,^26,45,46^ 10 Aβ+ patients met the diagnostic criteria for lvPPA^27^ and 2 Aβ+ patients met the criteria for dysexecutive Alzheimer’s disease.^28^ Participants also underwent neuroimaging sessions which included a high-resolution 3 T MRI, resting-state functional MRI (rs-fMRI), ^18^F-AV-1451 Tau PET and Pittsburgh Compound B (PiB) PET (amyloid PET) imaging. We selected patients for this study who had a positive amyloid PET scan, as assessed by visual read according to previously published procedures,^15^ and who met criteria for probable Alzheimer’s disease with a PetSurfer FLR distribution volume ratio >1.2.^47^ We chose to analyse a combined sample of PCA, lvPPA and dysexecutive Alzheimer’s disease patients to leverage the natural heterogeneity and range in cortical tau signal across patients, which allowed us to investigate the relationships between tau PET signal and resting-state functional connectivity in our networks of interest.

**Table 1:** Clinical characteristics of the Aβ+ Alzheimer’s disease group

Demographics	All (*N* = 25)	PCA (*N* = 13)	lvPPA (*N* = 10)	Dysexecutive (*N* = 2)
Age (years)	68.3 ± 8	67.3 ± 9.1	70.8 ± 6.2	62.5 ± 7.7
Sex (M/F)	9/16	3/10	4/6	2/0
Education (years)	16.5 ± 2.4	16.8 ± 1.9	15.7 ± 2.9	19.0 ± 1.4
MoCA	16.4 ± 5.9	15.2 ± 5.1	18.8 ± 6.1	12.5 ± 9.2
CDR	CDR 0.5 (*N* = 16) CDR 1 (*N* = 9)	CDR 0.5 (*N* = 7) CDR 1 (*N* = 6)	CDR 0.5 (*N* = 9) CDR 1 (*N* = 1)	CDR 1 (*N* = 2)
CDR-SOB	3.7 ± 1.8	4.5 ± 1.6	2.4 ± 1.3	4.7 ± 0.4
PiB FLR DVR	1.9 ± 0.2	1.7 ± 0.3	1.7 ± 0.2	1.9 ± 0.2

MoCA, Montreal Cognitive Assessment; CDR, clinical dementia rating; SOB, sum of box scores; PiB FLR DVR, Pittsburgh Compound B fronto-lateral-retrosplenial distribution value ratio.

We included a group of amyloid-negative cognitively normal (CN) individuals who all performed within normal limits on neuropsychological testing, had normal brain structure based on MRI and low cerebral amyloid based on quantitative analysis of amyloid PET distribution value ratio (DVR *<* 1.2), resulting in a CN sample of 25 individuals (Aβ− CN; mean age = 67.1 ± 4.7 years, 13M/12F). This Aβ− CN group was used primarily for comparisons with the Aβ+ Alzheimer’s disease group on tau PET measures. The second group of 30 CN individuals with unknown amyloid-beta status (CN2; mean age = 67.7 ± 4.6 years, 15M/15F) with rs-fMRI connectivity data were examined for comparison to the Aβ+ Alzheimer’s disease group on functional connectivity measures of interest. To define target regions of interest (ROIs) within the DMN, we also utilized resting-state data from a large group (*n* = 89) of healthy young controls (YCs).

Individuals were excluded from our patient and control cohorts if they had a primary psychiatric or other neurological disorder including major cerebrovascular infarct or stroke, seizure, brain tumour, hydrocephalus, multiple sclerosis, HIV-associated cognitive impairment or acute encephalopathy. This work was carried out in accordance with The Code of Ethics of the World Medical Association (Declaration of Helsinki) for experiments involving humans. All participants and their informants/caregivers provided informed consent in accordance with the protocol approved by the Mass General Brigham HealthCare System Human Research Committee Institutional Review Board in Boston, MA, USA.

### Cognitive screening

Twenty-five Aβ+ Alzheimer’s disease patients underwent a cognitive screen to better characterize their stage of disease severity pertaining to cognitive decline. All were administered the Montreal Cognitive Assessment (MoCA), a brief screening tool for cognitive impairment covering domains of orientation, executive functions, visuospatial cognition, memory and language.^48^ The total score on this test was calculated out of a maximum of 30; the means and standard deviation of our patient sample are included in Table 1. To determine the relationship between functional connectivity measures of interest and cognitive ability, bivariate correlation analysis was conducted between the total MoCA score and the Fisher-transformed functional connectivity *z*-score.

### Neuroimaging data acquisition and analysis

Twenty-five Aβ+ Alzheimer’s disease patients, 30 CN2 participants and 89 YC participants underwent an rs-fMRI scan (Siemens TIM Trio 3.0 T). This fMRI acquisition and preprocessing protocol was identical for the Aβ+ Alzheimer’s disease patients and 30 CN2 individuals and is described in detail in a previous publication.^49^ Images were acquired for 6.4 min during rest using a gradient-echo, echo-planar sequence with the following parameters: repetition time = 5000 ms, echo time = 30 ms, flip angle = 90°, one run of 76 TRs; voxel resolution: 2 mm isotropic. For the healthy YC group, this protocol was slightly different and consisted of 124 acquisitions (TR 3000 ms and T3 of 30 ms). During each functional MRI run, participants were directed to keep their eyes open and to remain as still as possible. Head motion was minimized using head restraints, including a pillow and foam padding. Noise was attenuated with earplugs. All rs-fMRI data were first preprocessed using the following steps: removal of the first four functional volumes to allow for T1 equilibration effects, slice timing correction (SPM2, Wellcome Department of Cognitive Neurology, London, UK), head motion correction using rigid-body transformation in three translations and three rotations (FMRIB, Oxford, UK), spatial normalization to standard (MNI 152) space, resampling to 2 mm isotropic voxels, spatial smoothing with a 6 mm FWHM Gaussian kernel and low-pass temporal filtering to remove frequencies >0.08 Hz. Sources of spurious variance and their temporal derivatives were removed from the preprocessed data using linear regression. These sources of spurious variance included the six parameters derived from head motion correction, the signal averaged over the whole brain, the average signal from a region located deep in white matter and the average signal from ventricular CSF. We further calculated the framewise displacement (FD) for each participant with a cut-off of 0.5 mm to detect individual BOLD volumes exhibiting excess motion.^50,51^ We found that one lvPPA participant and one CN2 participant had the majority of their BOLD volumes identified as showing excessive motion (58.3 and 51.4%, respectively). To ensure our results were robust to individual variation in the degree of motion, we repeated all analyses controlling for mean FD and found the same results. Further, we conducted all analyses without these two subjects included and again found the same results, reassuring us that excess motion did not impact the results or interpretability of our observations.

All 25 Aβ+ Alzheimer’s disease patients and 25 Aβ− CN control participants underwent ^18^F-AV-1451 (tau) PET scans. The ^18^F-AV-1451 radiotracer was prepared at MGH with a radiochemical yield of 14 ± 3% and specific activity of 216 ± 60 GBq/µmol (5837 ± 1621 mCi/µmol) at the end of synthesis (60 min) and validated for human use.^52^ Scans were acquired from 80 to 100 min after a 10.0 ± 1.0 mCi bolus injection in 4 × 5 min frames. All PET data were acquired using a Siemens/CTI (Knoxville, TN, USA) ECAT HR+ scanner (3D mode; 63 image planes; 15.2 cm axial field of view; 5.6 mm transaxial resolution and 2.4 mm slice interval). Data were reconstructed and attenuation corrected; each frame was evaluated to verify adequate count statistics; the interframe head motion was corrected prior to further processing. No data were excluded because of excess motion. All 25 Aβ+ Alzheimer’s disease patients and 25 Aβ− CN participants also underwent a high-resolution MRI scan (Siemens TIM trio 3.0 T) and tau (^18^F-AV-1451) PET imaging. The MRI scan session included the acquisition of T1-weighted multi-echo magnetization prepared rapid acquisition gradient-echo (MPRAGE) structural images. Visual inspection confirmed accurate registration between anatomical and PET volumes. To evaluate the anatomy of PET binding, each individual’s PET data set was rigidly co-registered to the subject’s MPRAGE data using the FreeSurfer *mri_coreg* command. Similar to a previous report, ^18^F-AV-1451 specific binding was expressed as the standardized uptake value ratio (SUVR) using the whole cerebellar GM as a reference.^53^ PET data were normalized to an MNI template using the ANTS (Advanced Normalization Tools) registration method. PET data were also partial volume-corrected, performed using geometric transform matrix as implemented in FreeSurfer stable release version 6.0.

Structural MRI data were analysed with FMRIB Software Library (FSL)-voxel-based morphometry (VBM),^54^http://fsl.fmrib.ox.ac.uk/fsl/fslwiki/FSLVBM, an optimized VBM protocol,^55^ carried out with FSL tools.^56^ Images were first preprocessed by reorienting the scan, skull stripping and segmenting the tissue. The resulting GM partial volume maps were then registered to the MNI152 standard template and then registered to a study-specific template, combining Aβ+ Alzheimer’s disease and Aβ− CN images, to which the native GM images were re-registered. The resulting partial volume maps were then modulated by dividing them by the Jacobian of the warp field. Finally, the modulated images were smoothed with an isotropic Gaussian kernel with a sigma of 3 mm (full width half max of 8 mm). The average Jacobian values from our ROIs listed previously were extracted from the resulting VBM maps and utilized in bivariate correlational analyses to determine the relationship between GM density in our ROIs and functional connectivity within our networks of interest.

**Figure 1:**
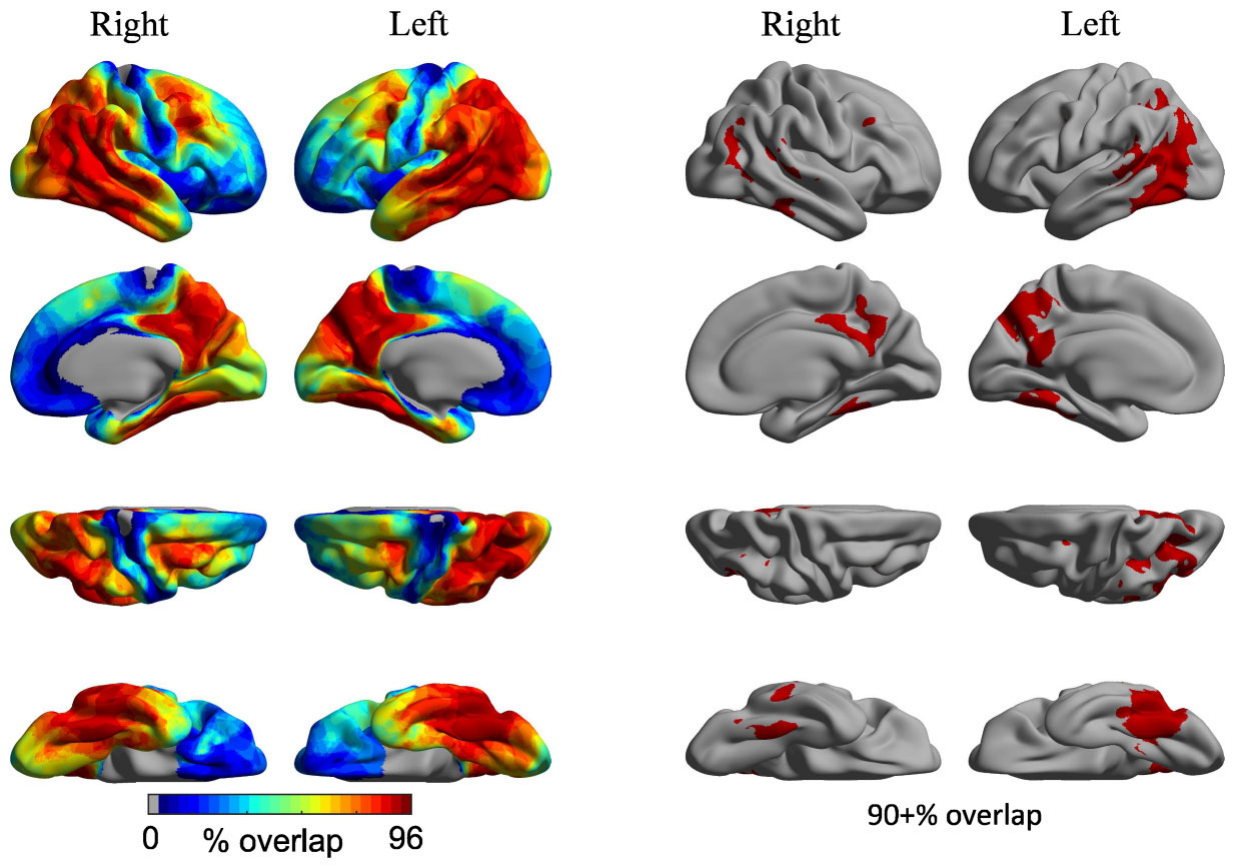
**Individual variability in the cortical distribution of tau PET signal.** Coloured vertices on the cortical surface map indicate the degree of spatial overlap in elevated tau across individual patients. Raw *W*-score maps were binarized at *W* > 3, corresponding to the 99th percentile of the normal distribution, summed across all patients, divided by the number of patients and multiplied by 100. The overlap map thus identifies the extent of between-subjects consistency in elevated tau uptake (from 0 to 100%). All patients exhibited elevated tau in the posterior DMN, including the PCC and lateral temporoparietal cortex.

To determine if the majority of our atypical Alzheimer’s disease group demonstrated elevated tau PET signal in the posterior DMN nodes hypothesized, vertex-wise SUVR values were converted to *W*-scores (i.e. *z*-scores adjusted for age and sex based on healthy controls). Elevated tau PET signal was defined as *W* > 3, corresponding to the 99th percentile of the normal distribution.^57^ Binarized *W*-score maps were created using this threshold and were summed across all patients. Once converted to percentage, this overlap map (Fig. 1) identifies, at each cortical vertex, the extent of between-subject consistency in elevated tau PET signal.

### Defining ROIs

ROIs were largely derived from our YC group resting-state connectivity maps, although we began by defining 5 m spherical ROIs from key coordinates of the PCC from the literature^58^: left PCC [−8 −56 26] and right PCC [8 −56 26]. All ROI coordinates are reported as MNI coordinates. Whole brain functional connectivity maps were generated from these two seed ROIs in the YC group to determine the other most highly connected regions comprising the DMN, bounded by *a priori* ROIs using the FSL Harvard–Oxford cortical atlas. The other DMN ROIs were defined as the left [−46 −68 36] and right [50 −64 30] angular gyrus (AG), left [−60 −10 −22] and right [60 −6 −24] lateral temporal cortex (LTC), left [−22 −20 −24] and right [24 −18 −24] hippocampal formation (Hipp) and left [−4 52 −6] and right [4 52 −6] anterior medial prefrontal cortex (amPFC). ROI-to-ROI connectivity analysis was then assessed between these DMN ROIs within each hemisphere and defined as the mean Pearson’s product-moment correlation ‘*r*’ converted to *z*-scores using Fisher’s *r*-to-*z* transformation between any two ROIs. Partial volume-corrected tau PET SUVR values were also extracted from these same ROIs (PCC, AG, LTC, Hipp and amPFC) in each hemisphere. These same five spherical ROIs were also used in VBM analyses to calculate GM density.

### Statistical analyses

Resting-state fcMRI comparisons between the Aβ+ Alzheimer’s disease group and the CN2 group, as well as tau PET SUVR comparisons between the Aβ+ Alzheimer’s disease group and the Aβ− CN group were conducted using independent samples *t*-tests. To determine the relationship between tau PET SUVR and functional connectivity, we conducted bivariate correlational analyses between tau PET SUVR in our ROIs and the Fisher *z*-transformed functional connectivity values from our ROI-to-ROI analysis in the atypical Alzheimer’s disease group (Supplementary Table 1). A similar correlational analysis was conducted using VBM Jacobian values to determine if GM density in our ROIs was related to the Fisher *z*-transformed functional connectivity values between our DMN ROIs in the atypical Alzheimer’s disease group. Hierarchical regression analysis was then performed to determine if tau PET SUVR values were predictive of functional connectivity over and above GM density. Based on previous work in typical amnestic Alzheimer’s disease suggesting both a decrease in DMN connectivity specifically in the PCC^59^ and that tau spreads between regions with the highest functional connectivity to each other,^21^ we specifically approached these correlation analyses with *a priori* hypotheses that tau in the two highly synchronous posterior DMN nodes, PCC and AG, would be related to functional connectivity between these same regions. We secondarily conducted exploratory analyses between tau and functional connectivity in the other ROIs within the DMN.

### Data availability statement

The data that support the findings of this study are available from the corresponding author, upon reasonable request.

## Results

### Clinical characteristics of the atypical Aβ+ Alzheimer’s disease group

Table 1 shows the clinical characteristics of the Aβ+ Alzheimer’s disease group. With regard to cognitive functional status, the patients included in this study were either classified as having a mild cognitive impairment in the clinical dementia rating (CDR = 0.5) or mild dementia (CDR = 1), with the majority of participants (16/25) in this study rated at the stage of mild cognitive impairment.

### High overlap of Tau PET signal in the posterior but not anterior DMN across the Aβ+ atypical Alzheimer’s disease spectrum

To investigate whether the PCC and other regions of the posterior DMN are indeed hub regions of tau deposition in Aβ+ atypical Alzheimer’s disease phenotypes, we conducted a *W*-score analysis. Elevated tau PET signal was defined as *W* > 3, corresponding to the 99th percentile of the normal distribution. Individual binarized *W*-score maps were created using this threshold and were summed across all patients. This overlap map identifies, at each cortical vertex, the extent of between-subject consistency in elevated tau PET signal across all 25 Aβ+ Alzheimer’s disease participants. We found that bilateral PCC, along with precuneus and bilateral temporoparietal cortex, showed 90% or higher tau SUVR overlap across all participants (Fig. 1).

**Figure 2:**
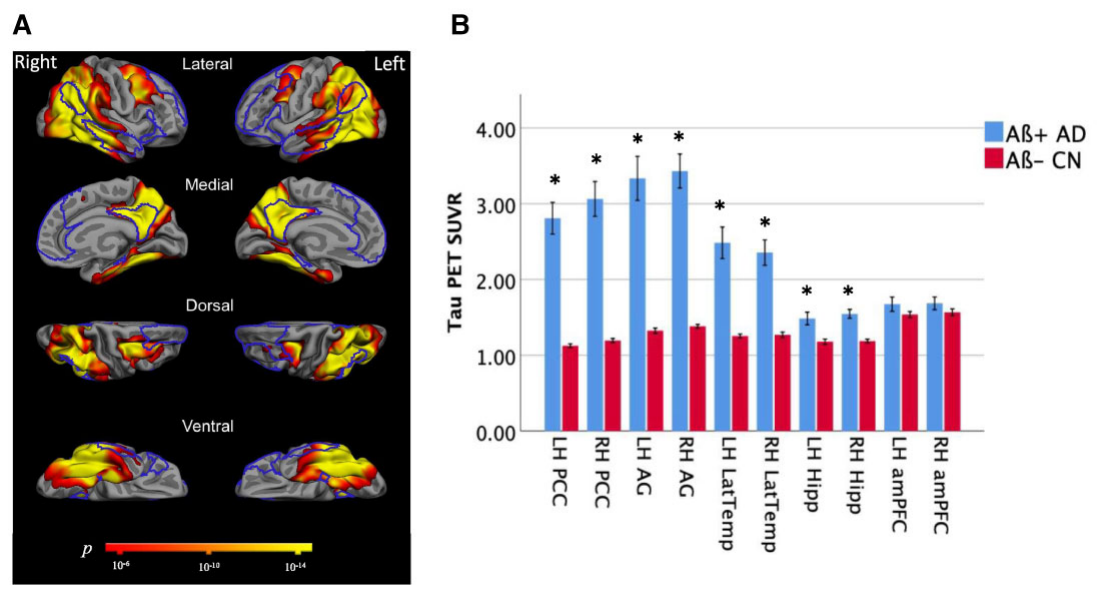
**Mean tau PET signal is high across the posterior DMN in Aβ+ Alzheimer’s disease.** (**A)** Maps of the mean cortical tau (^18^F-AV-1451) PET in 25 Aβ+ Alzheimer’s disease patients compared with 25 Aβ− CN participants, with significance thresholded at *P* < 10^−6^. The DMN is outlined. (**B)** Mean partial volume-corrected tau PET SUVR values in ROIs comprising the DMN were compared between the Aβ+ Alzheimer’s disease patients and Aβ− CN group with independent samples *t*-tests. Elevated tau signal in the left (*t* = 8.0, *P* = 2.5 × 10^−10^) and right (*t* = 8.1, *P* = 1.7 × 10^−10^) PCC, left (*t* = 6.9, *P* = 1.2 × 10^−8^) and right (*t* = 9.05, *P* = 6.0 × 10^−12^) AG, left (*t* = 5.9, *P* = 4.2 × 10^−7^) and right (*t* = 6.3, *P* = 7.6 × 10^−8^) LTC and left (*t* = 3.4, *P* = 0.001) and right (*t* = 5.6, *P* = 9.2 × 10^−7^) hippocampal formation were observed in the Aβ+ Alzheimer’s disease patients compared with Aβ− CN participants. PCC, posterior cingulate cortex; AG, angular gyrus; LatTemp, lateral temporal cortex; Hipp, hippocampus; LH, left hemisphere; RH, right hemisphere. *Significant group difference at the level of *P* < 0.005. There were no group differences in tau PET SUVR values in the left and right amPFC. SUVR, standard uptake value ratio; ROI, region of interest.

Furthermore, compared with amyloid-negative age-matched control participants (Aβ− CN), whole brain maps of cortical tau in the Aβ+ atypical Alzheimer’s disease participants as a group revealed widespread tau deposition in regions of medial and lateral temporal cortices, medial and lateral parietal cortices including the PCC and precuneus and regions of the dorsolateral prefrontal cortex (Fig. 2A). Partial volume-corrected tau PET SUVR was elevated in bilateral posterior but not anterior DMN ROIs examined in the atypical Aβ+ Alzheimer’s disease group compared with the Aβ− CN group (Fig. 2B): left PCC: *t* = 8.0, *P* = 2.5 × 10^−10^; right PCC: *t* = 8.1, *P* = 1.7 × 10^−10^; left AG: *t* = 6.9, *P* = 1.2 × 10^−8^; right AG: *t* = 9.05, *P* = 6.0 × 10^−12^; left LatTemp: *t* = 5.9, *P* = 4.2 × 10^−7^; right LatTemp: *t* = 6.3, *P* = 7.6 × 10^−8^; left Hipp: *t* = 3.4, *P* = 0.001; right Hipp: *t* = 5.6, *P* = 9.2 × 10^−7^.

### Functional hypoconnectivity between temporal and parietal nodes of the DMN in Aβ+ Alzheimer’s disease

**Figure 3:**
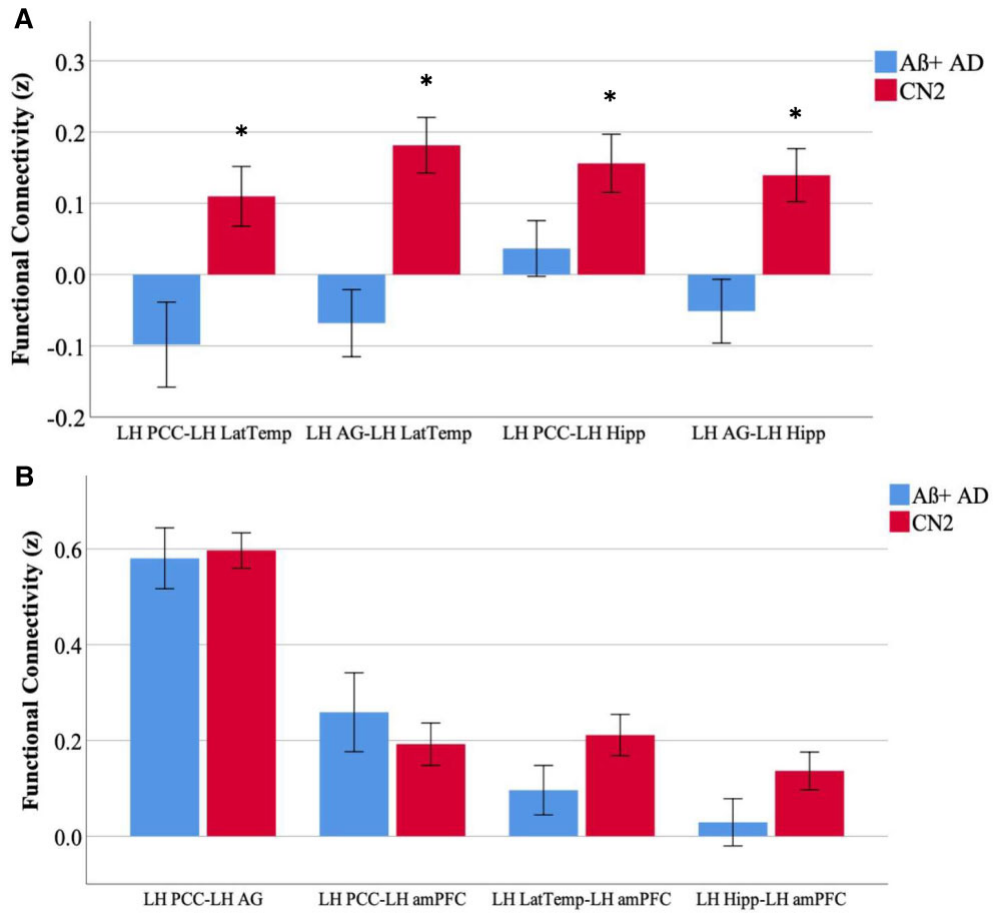
**Functional hypoconnectivity between temporal and parietal nodes of the DMN**. Independent samples *t*-tests were conducted to determine the group mean differences between the Aβ+ Alzheimer’s disease and CN2 groups in functional connectivity (Fisher’s *z*-score) between DMN ROIs. (**A)** Functional hypoconnectivity in the Aβ+ Alzheimer’s disease group was observed between the left PCC and LatTemp (*t* = 2.9, *P* = 0.005), between the left AG and left LatTemp (*t* = 4.1, *P* = 0.0001), between the left PCC and left Hipp (*t* = 2.1, *P* = 0.04) and between the left AG and left Hipp (*t* = 3.3, *P* = 0.002). (**B)** Functional connectivity was comparable between posterior parietal nodes (PCC and AG), between parietal and frontal nodes (PCC and amPFC) and between temporal and parietal nodes (LatTemp, Hipp and amPFC). PCC, posterior cingulate cortex; LatTemp, lateral temporal cortex; AG, angular gyrus. Hipp, Hippocampus; amPFC, anterior medial prefrontal cortex; LH, left hemisphere. *Significance at the level of *p* < 0.05. Functional connectivity between left-hemisphere ROIs is shown here for illustrative purposes; complete group differences are described in the text.

Comparing functional connectivity in the Aβ+ Alzheimer’s disease group compared with the control group (Fig. 3 and Supplementary Fig. 1), we found functional hypoconnectivity between the temporal nodes of the DMN (LatTemp and Hipp) and the parietal nodes of the DMN (PCC and AG). Specifically, we observed hypoconnectivity in our Aβ+ Alzheimer’s disease patients (Fig. 3A) between the left LatTemp and PCC (*t* = 2.9, *P* = 0.005), between the left LatTemp and AG (*t* = 4.1, *P* = 0.0001), between the right LatTemp and AG (*t* = 3.5, *P* = 0.001), between the left Hipp and PCC (*t* = 2.1, *P* = 0.04), between the left Hipp and AG (*t* = 3.3, *P* = 0.002) and between the right Hipp and AG (*t* = 2.1, *P* = 0.04). We did not observe any between-group differences in functional connectivity between the PCC and AG, between parietal and frontal ROIs or between temporal and frontal ROIs in either hemisphere (Fig. 3B).

### Tau is related to functional hypoconnectivity between the posterior parietal nodes

**Figure 4:**
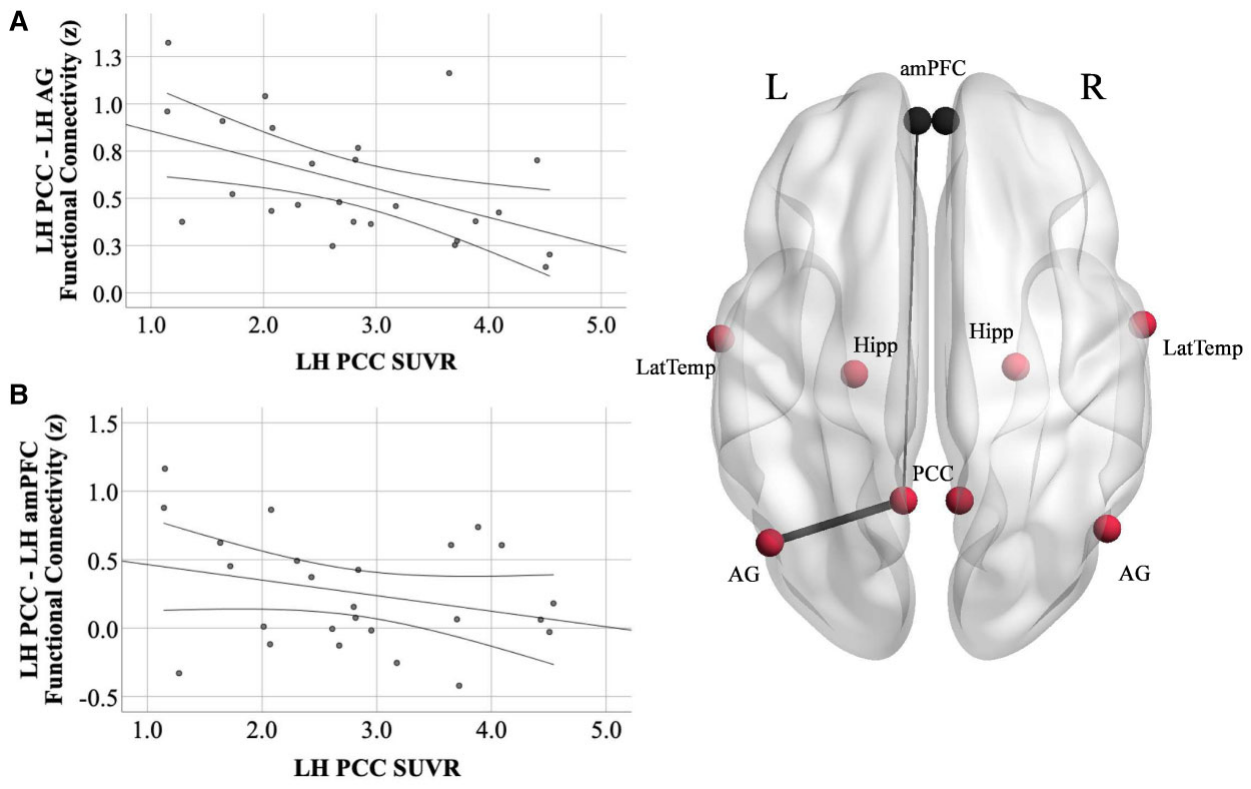
**Tau PET SUVR in the PCC is related to PCC-to-AG hypoconnectivity but not PCC-amPFC connectivity.** (**A**) Increased tau PET signal in the left PCC is related to reduced functional connectivity between the left PCC and AG (*r* = −0.51, *P* = 0.01) in Aβ+ Alzheimer’s disease. (**B**) There was no relationship between tau PET signal in the left PCC and functional connectivity between the left PCC and amPFC (*r* = −0.29, *P* = 0.2). PCC, posterior cingulate cortex; AG, angular gyrus; amPFC, anterior medial prefrontal cortex; LH, left hemisphere. Functional connectivity units are Fisher’s *z*-scores. 95% confidence intervals are displayed. Results from the left hemisphere are shown here for illustrative purposes; complete results from both hemispheres are described in the text.

We conducted bivariate correlations between tau PET SUVR in the posterior parietal DMN nodes (PCC and AG) and functional connectivity between these regions in each hemisphere according to our specific *a priori* hypotheses regarding the relationship between these two modalities. Consistent with our hypotheses, we found a negative relationship between tau in the LH PCC and functional connectivity in the left posterior parietal DMN (PCC-to-AG; *r* = −0.50, *P* = 0. 01; Fig. 4A and Supplementary Fig. 2A), such that increased left PCC tau was related to reduced functional connectivity between the left PCC and AG. Tau PET SUVR values in the PCC and AG were highly correlated with each other (*r* > 0.8) in each hemisphere. Thus, we observed similar relationships between left PCC-to-AG connectivity and right PCC tau (*r* = −0.43, *P* = 0.03), left AG tau (*r* = −0.46, *P* = 0.02) and right AG tau (*r* = −0.46, *P* = 0.02). Neither PCC tau nor AG tau in either hemisphere was related to right PCC-to-AG functional connectivity. Secondarily, we conducted exploratory analyses between tau in the posterior parietal DMN and functional connectivity with other ROIs within the DMN. In a clear dissociation, we did not observe relationships between tau and functional connectivity between posterior parietal ROIs (PCC and AG) and temporal ROIs (LatTemp, Hipp), though functional hypoconnectivity between these parietal and temporal ROIs was found in patients compared with controls. We also did not observe any relationship between tau in posterior parietal ROIs and functional connectivity between posterior parietal ROIs and the anterior ROI of the DMN, the amPFC, in either hemisphere (Fig. 4B and Supplementary Fig. 2B).

### GM density is related to tau and functional connectivity in the posterior parietal cortex

To determine if the relationships we observed between tau and functional connectivity within the posterior parietal cortex were related in part to cortical volume loss, we also investigated the relationship between GM density and tau. We also examined the relationship between GM density and functional connectivity between the PCC and AG. As expected, we found that tau SUVR and GM density were negatively correlated with each other in the left PCC (*r* = −0.47, *P* = 0.02) and the right PCC (*r* = −0.64, *P* = 0.001). Next, we found that the GM density of the left PCC and right PCC were also related to left PCC-to-AG connectivity (left PCC: *r* = 0.56, *P* = 0.004; right PCC: *r* = 0.42, *P* = 0.03). We did not observe a relationship between the GM density of the AG in either hemisphere and PCC-to-AG functional connectivity.

When left PCC GM density was added as an independent predictor in addition to left PCC tau SUVR in linear regression models where the dependent variable was left PCC-to-AG functional connectivity, tau was no longer an independent predictor of functional connectivity suggesting that the relationship between tau and functional connectivity may be mediated by GM density. A formal mediation analysis did not yield significant results (Sobel test = −1.55, *P* = 0.12), though we may be underpowered to detect these effects.

### Functional hypoconnectivity is related to poorer cognition

To determine if the functional hypoconnectivity observed between posterior DMN nodes was related to a cognitive decline (as measured by the MoCA), we conducted bivariate correlations between the functional connectivity *z*-scores and total raw MoCA scores in our Aβ+ Alzheimer’s disease group. Specifically, the MoCA total score was related to functional connectivity between the left PCC and left AG (*r* = 0.49, *P* = 0.01), as well as between left PCC and left LatTemp (*r* = 0.44, *P* = 0.03).

## Discussion

Functional disconnection within the DMN has been consistently reported in Alzheimer’s disease,^7–10,59^ and hypoconnectivity within the DMN has been related to biomarkers of Aβ and tau pathology.^11,12,20^ However, much of our understanding of the relationships between DMN connectivity and Alzheimer’s disease biomarkers has focused on older-onset Alzheimer’s disease patients with typical amnestic symptoms, which is only one part of the spectrum of the illness. Although the primary drivers of the earliest symptoms in atypical phenotypes of Alzheimer’s disease (e.g. PCA, lvPPA) are observed in other domain-specific networks supporting visuospatial cognition and language, respectively,^41^ we hypothesized that the DMN is also critically affected across the atypical Alzheimer’s disease phenotypic spectrum, given the high levels of atrophy^33–36^ and tau pathology^17,19,37^ reported in the posterior temporal and parietal regions of the DMN in atypical phenotypes of Alzheimer’s disease. To the best of our knowledge, no study to date has examined how tau pathology within the core nodes of the DMN relates to network connectivity across atypical phenotypes of Alzheimer’s disease. Here, we hypothesized that we would observe high levels of tau burden and hypoconnectivity in the posterior regions of the DMN in patients with atypical Alzheimer’s disease, and that increased tau in these regions would be related to reduced functional connectivity between posterior regions of the DMN.

We first identified areas of highest tau overlap across our sample in the medial and lateral parietal and lateral temporal cortices of both hemispheres, with left-hemisphere predominance (Fig. 1). The lateralization observed here may reflect the high number of lvPPA patients who make up 42% of our study sample. Further, we observed high tau signal across all posterior DMN ROIs (PCC, AG, LatTemp and Hipp) in patients compared with Aβ− age-matched controls in both hemispheres but no differences in the amPFC (Fig. 2). These observations are consistent with previous work identifying atrophy^33–36^ and tau pathology,^17,19,37^ particularly in the posterior temporoparietal regions of the DMN in early-onset atypical Alzheimer’s disease.

We next examined how functional connectivity within the DMN breaks down in atypical Alzheimer’s disease compared with healthy age-matched controls (Fig. 3). Consistent with prior work in typical, older-onset prodromal Alzheimer’s disease,^7,8,60,61^ we found evidence of functional hypoconnectivity between the temporal (LatTemp, Hipp) and parietal (PCC, AG) nodes of the DMN. We did not observe any group differences in functional connectivity between the PCC and AG, despite a high level of tau burden in both of these posterior ROIs. It is possible that the connectivity between the PCC and AG is resilient to decoupling as a result of high baseline connectivity between them.^38^ Critically, we also did not observe group differences between parietal and frontal ROIs (such as PCC and amPFC), or between temporal and frontal ROIs in either hemisphere. The frontal ROI examined in this study was the amPFC, a region that did not carry a high level of tau in this sample. This finding initially appears inconsistent with previous reports of reduced PCC-to-amPFC connectivity in symptomatic Alzheimer’s disease dementia patients.^10,60^ However, the patients in our atypical Alzheimer’s disease sample were largely at an earlier stage of disease progression (CDR = 0.5), and both these other studies and others^9,62,63^ report progressively weaker anterior-to-posterior DMN connectivity as disease severity progresses. These observations suggest that the connections between posterior and anterior nodes of the DMN may be preserved until later stages of the disease across clinical phenotypes of Alzheimer’s disease. Taken together, one interpretation of these results is that functional connectivity between the temporal and parietal regions of the DMN may become dysfunctional before intraparietal regions do. The PCC, inferior parietal lobule and retrosplenial cortex are densely interconnected with each other and have been described as forming a ‘structural core’ of cortical connections.^38^ This same study revealed that the medial prefrontal cortex was excluded from this structural core; the authors suggest that DMN activity may be driven from highly coupled areas of the posterior medial and parietal cortices. Future work examining the relative strength of functional connectivity between the major nodes of the DMN is required to support this interpretation. Finally, our observations that functional hypoconnectivity within the posterior ROIs (PCC-to-AG and PCC-to-Lat Temp) is related to poorer cognitive scores further support our conclusions that these deteriorations in functional connectivity within the DMN indicate disease progression in the relatively mild stage of Alzheimer’s disease.

We directly tested the relationship between tau pathology and functional connectivity within the DMN and found that higher tau in the PCC and AG is related to reduced functional connectivity between these two regions in the left hemisphere (Fig. 4A) despite no measurable loss of functional connectivity between the PCC and AG. This observation is consistent with recent work in early amnestic Alzheimer’s disease demonstrating that tau PET uptake with a different tracer (18-F THK5351) was related to decreased connectivity between the PCC/precuneus and widespread regions of the brain including the medial and lateral temporal lobes.^23^ Interestingly, a similar relationship has also been observed in DLB patients with concurrent Alzheimer’s disease pathology such that a reduction in posterior DMN connectivity correlated with overall higher cortical Alzheimer’s disease-related tau PET uptake.^24^ Exploratory *post hoc* analyses in another recent study suggested that tau in the precuneus/posterior DMN was specifically associated with declining posterior DMN connectivity and increasing frontal lobe connectivity across the symptomatic spectrum of typical Alzheimer’s disease.^64^ In the present study, we did not find a relationship between tau in the PCC and AG in either hemisphere and functional connectivity between the right-hemisphere PCC and AG. This may in part reflect baseline differences in the strength of functional connections between the hemispheres; one multimodal study in healthy young adults observed higher functional connectivity between midline PCC and right-hemisphere AG compared with the left-hemisphere AG,^65^ possibly reflecting greater vulnerability in the left-hemisphere functional connections between PCC and AG. Perhaps our observation in this study that increased tau is related to hypoconnectivity between the left-hemisphere PCC and AG and not between right-hemisphere PCC and AG reflects this left-hemisphere vulnerability between the parietal regions of the DMN. Additionally, as noted above, the lateralization may also reflect, in part, the composition of our study sample, which included a large number of lvPPA patients.

We did not observe any other relationships between tau and functional connectivity within the DMN, including between the PCC and amPFC (Fig. 4B). As discussed previously, this finding may reflect the fact that this sample consists of patients at relatively earlier stages of disease progression. We might expect to see this relationship between tau and anterior-to-posterior DMN connectivity as disease severity progresses.^9,62,63^ Together with our observation that increasing tau relates to decreasing functional connectivity between the PCC and AG, these results may indicate that the PCC and AG are in the process of disconnection, whilst PCC-amPFC connectivity has not yet been decoupled, perhaps in part due to low tau levels in the amPFC. This interpretation is supported by recent work suggesting that longitudinal tau spread is observed most prominently in the frontal cortex as the disease progresses in atypical^37^ and typical Alzheimer’s disease,^66^ rather than in the posterior cortical regions typically impacted first in Alzheimer’s disease which may be saturated with tau over time. Thus, we observe a fractionated DMN in atypical Alzheimer’s disease at this stage of the disease course. Based on the work of Andrews-Hanna *et al*.^58^ which introduced the idea of DMN subsystems, our cross-sectional results could indicate that the dorsomedial subsystem (which includes Lat Temp) and medial temporal subsystem (which includes hippocampus and AG) of the DMN may decouple from the posterior node of the core DMN network (PCC) before regions comprising the core DMN network decouple from each other (PCC and amPFC). Longitudinal studies of the relationships between tau accumulation and functional connectivity changes over time are needed to clarify this hypothesized timeline of DMN fractionation in atypical Alzheimer’s disease.

Finally, we observed a strong relationship between reduced GM density in the left- and right-hemisphere PCC and functional connectivity between the left-hemisphere PCC and AG, which follows logically from our observations that tau uptake was strongly correlated with volume loss in the PCC. Tau PET uptake has been closely related to cortical volume loss across the syndromic spectrum of Alzheimer’s disease,^19,67,68^ although they potentially reflect different contributions to clinical decline. Specifically, volume loss may be a consequence of several neurological processes, including a contribution from Alzheimer’s disease-related pathologies such as tau and Aβ but also from vascular and other co-existing pathologies in older adults.^67,69^ This, together with the observation that structural volume loss in Alzheimer’s disease is closely related to neuropathologically defined tau deposition in Alzheimer’s disease,^70–72^ led us to investigate how tau and GM loss interact in relation to functional connectivity changes. Although we hypothesized that volume loss would partially mediate the relationship between tau and functional connectivity changes, we did not observe this effect in formal mediation analysis. This may be due to our relatively small sample size being underpowered to detect such relationships. It is possible that tau is driving the functional hypoconnectivity and contributing to, but not fully causing, volume loss in these same regions.

Our study had some limitations that are important to recognize. First, as noted above, we had a relatively small sample size, which may have underpowered our analyses detecting the independent effects of tau and GM density, and particularly with our formal mediation analysis. Reassuringly, the relationships between tau and functional connectivity, as well as between volume loss and functional connectivity, were robust and consistent with previous observations in typical older-onset amnestic Alzheimer’s disease. As these atypical Alzheimer’s disease populations are rare, it will be important for these results to be replicated in a larger sample through multi-centre collaborative efforts. Along these lines, larger and more homogenous atypical Alzheimer’s disease subgroups will be important to study to determine if these observations are particular to any single syndromic presentation. Finally, another limitation is the cross-sectional nature of this study; our results suggest a possible timeline of functional network degradation within the DMN which should be evaluated with a longitudinal study design.

In summary, we demonstrate that the DMN fractionates in patients with atypical clinical presentations of Alzheimer’s disease who are mostly at the stage of mild cognitive impairment, largely consistent with previous observations in typical older-onset amnestic presentations, such that the temporal and parietal nodes are no longer functionally connected with each other. We provide novel evidence that higher levels of tau pathology in posterior parietal nodes of the DMN (PCC, AG) are related to decreased connectivity between these parietal nodes, which may reflect functional disconnection in the process. We also found that several nodes remain strongly functionally connected to each other comparable to healthy controls, including parietal to frontal regions comprising the ‘core’ DMN, which suggests that regions with low tau (i.e. amPFC) are likely to retain functional connections with other regions with the DMN which carry a high tau burden (e.g. PCC) until later stages of the disease.

## Supplementary Material

fcac055_Supplementary_Data

## Abbreviations

Aβ+ =: amyloid-beta positive
AG =: angular gyrus
amPFC =: anterior medial prefrontal cortex
CN =: cognitively normal
DMN =: default mode network
FSL =: FMRIB Software Library
GM =: grey matter
Hipp =: hippocampus
LatTemp =: lateral temporal cortex
lvPPA =: logopenic variant primary progressive aphasia
MGH =: Massachusetts General Hospital
MNI =: Montreal Neurological Institute
MoCA =: Montreal Cognitive Assessment
MTL =: medial temporal lobe
PCA =: posterior cortical atrophy
PCC =: posterior cingulate cortex
ROI =: region of interest
SPM =: Statistical Parametric Mapping
SUVR =: standard uptake value ratio
VBM =: voxel-based morphometry
