# Supplementary material for: Tau and the fractionated default mode network in atypical Alzheimer’s disease
Source: Brain Commun. 2022 Mar 9;4(2):fcac055. doi: 10.1093/braincomms/fcac055 (PMC8963312; doi:10.1093/braincomms/fcac055)
Supplement: fcac055_Supplementary_Data [file fcac055_supplementary_data.docx]

Supplementary Material

**Supplementary Table 1. Tau in relation to functional connectivity within the DMN.** Bivariate correlations were conducted to determine the relationship between Tau PET SUVR in DMN ROIs and the ROI-to-ROI connectivity in each hemisphere. * indicates significance at the level of *p*<0.05.

| **Tau SUVR** | **ROI-to-ROI Functional Connectivity (z)** |  | **Tau SUVR** | **ROI-to-ROI Functional Connectivity (z)** |
| --- | --- | --- | --- | --- |
|  | LH PCC-to-LH AG |  |  | LH AG-to-LH Hipp |
| LH PCC Tau | ***r*= -0.50, *p*= 0.01*** |  | LH AG Tau | *r*= -0.16, *p*= 0.44 |
| RH PCC Tau | ***r*= -0.43, *p*=0.03*** |  | RH AG Tau | *r*= 0.21, *p*= 0.31 |
| LH AG Tau | ***r*= -0.46, *p*= 0.02*** |  | LH Hipp Tau | *r*= -0.17, *p*= 0.43 |
| RH AG Tau | ***r*= -0.46, *p*= 0.02*** |  | RH Hipp Tau | *r*= -0.05, *p*= 0.80 |
|  | RH PCC-to-RH AG |  |  | RH AG-to-RH Hipp |
| LH PCC Tau | *r*= -0.009, *p*= 0.96 |  | LH AG Tau | *r*= 0.34, *p*= 0.09 |
| RH PCC Tau | *r*= -0.043, *p*= 0.84 |  | RH AG Tau | *r*= 0.33, *p*= 0.11 |
| LH AG Tau | *r*= -0.19, *p*= 0.37 |  | LH Hipp Tau | *r*= 0.33, *p*= 0.11 |
| RH AG Tau | *r*= -0.07, *p*= 0.75 |  | RH Hipp Tau | *r*= 0.30, *p*= 0.16 |
|  | LH PCC-to-LH LatTemp |  |  | LH PCC-to-LH amPFC |
| LH PCC Tau | *r*= -0.11, *p*= 0.60 |  | LH PCC Tau | *r*= -0.29, *p*= 0.16 |
| RH PCC Tau | *r*= -0.20, *p*= 0.35 |  | RH PCC Tau | *r*= -0.31, *p*= 0.14 |
| LH LatTemp Tau | *r*= -0.29, *p*= 0.16 |  | LH amPFC Tau | *r*= -0.08, *p*= 0.71 |
| RH LatTemp Tau | *r*= -0.46, *p*= 0.06 |  | RH amPFC Tau | *r*= -0.24, *p*= 0.24 |
|  | RH PCC-to-RH LatTemp |  |  | RH PCC-to-RH amPFC |
| LH PCC Tau | *r*= 0.16, *p*= 0.44 |  | LH PCC Tau | *r*= 0.13, *p*= 0.54 |
| RH PCC Tau | *r*= 0.11, *p*= 0.60 |  | RH PCC Tau | *r*= 0.14, *p*= 0.50 |
| LH Lat Temp Tau | *r*= 0.30, *p*= 0.15 |  | LH amPFC Tau | *r*= 0.11, *p*= 0.60 |
| RH Lat Temp Tau | *r*= 0.11, *p*= 0.61 |  | RH amPFC Tau | *r*= -0.03, *p*= 0.88 |
|  | LH AG-to-LH LatTemp |  |  | LH LatTemp-to-LH amPFC |
| LH AG Tau | *r*= -0.07, *p*= 0.73 |  | LH LatTemp Tau | *r*= -0.37, *p*= 0.07 |
| RH AG Tau | *r*= 0.002, *p*= 0.99 |  | RH LatTemp Tau | *r*= -0.42, *p*= 0.06 |
| LH LatTemp Tau | *r*= -0.21, *p*= 0.31 |  | LH amPFC Tau | *r*= -0.41, *p*= 0.05 |
| RH LatTemp Tau | *r*= -0.18, *p*= 0.39 |  | RH amPFC Tau | *r*= -0.33, *p*= 0.11 |
|  | RH AG-to-RH LatTemp |  |  | RH LatTemp-to-RH amPFC |
| LH AG Tau | *r*= 0.20, *p*= 0.34 |  | LH LatTemp Tau | *r*= 0.09, *p*= 0.66 |
| RH AG Tau | *r*= 0.25, *p*= 0.22 |  | RH LatTemp Tau | *r*= -0.25, *p*= 0.22 |
| LH LatTemp Tau | *r*= 0.09, *p*= 0.66 |  | LH amPFC Tau | *r*= -0.12, *p*= 0.58 |
| RH LatTemp Tau | *r*= 0.18, *p*= 0.38 |  | RH amPFC Tau | *r*= -0.26, *p*= 0.22 |
|  | LH PCC-to-LH Hipp |  |  | LH Hipp-to-LH amPFC |
| LH PCC Tau | *r*= -0.21, *p*= 0.32 |  | LH Hipp Tau | *r*= -0.28, *p*= 0.18 |
| RH PCC Tau | *r*= -0.10, *p*= 0.64 |  | RH Hipp Tau | *r*= -0.02, *p*= 0.92 |
| LH Hipp Tau | *r*= -0.25, *p*= 0.23 |  | LH amPFC Tau | *r*= -0.23, *p*= 0.27 |
| RH Hipp Tau | *r*= -0.06, *p*= 0.78 |  | RH amPFC Tau | *r*= -0.18, *p*= 0.38 |
|  | RH PCC-to-RH Hipp |  |  | RH Hipp-to-RH amPFC |
| LH PCC Tau | *r*= 0.08, *p*= 0.69 |  | LH Hipp Tau | *r*= -0.27, *p*= 0.19 |
| RH PCC Tau | *r*= 0.24, *p*= 0.25 |  | RH Hipp Tau | *r*= -0.32, *p*= 0.12 |
| LH Hipp Tau | *r*= 0.25, *p*= 0.23 |  | LH amPFC Tau | *r*= -0.36, *p*= 0.08 |
| RH Hipp Tau | *r*= 0.17, *p*= 0.42 |  | RH amPFC Tau | *r*= -0.38, *p*= 0.06 |

**Supplementary Figure 1. Results from manuscript Figure 3 are displayed here with individual values plotted and Aß+ AD phenotypes identified: Functional hypoconnectivity between temporal and parietal nodes of the DMN. SM 1A.** Functional hypoconnectivity was observed between temporal (LatTemp and Hipp) and posterior parietal nodes (PCC, AG) of the DMN*.* **SM 1B.** Functional connectivity was comparable between the Aß+ AD and CN2 groups between posterior parietal nodes (PCC and AG), between parietal and frontal nodes (PCC and amPFC), and between temporal and parietal nodes (LatTemp, Hipp and amPFC). Functional connectivity (Fisher z-score) between left hemisphere ROIs is shown here for illustrative purposes; complete group differences are described in the text. LH = left hemisphere.

**Supplementary Figure 2. Results from manuscript Figure 4 are displayed here with Aß+ AD phenotypes identified: Tau PET SUVR in the PCC is related to PCC-to-AG hypoconnectivity but not PCC-amPFC connectivity. SM 2A.** Increased tau PET signal in the left PCC is related to reduced functional connectivity between left PCC and AG (*r*= -0.51, *p*=0.01) in Aß+ AD. **SM 2B.** There was no relationship between tau PET signal in the left PCC and functional connectivity between the left PCC and amPFC (*r=*-0.29, *p*= 0.2). PCC = posterior cingulate cortex. AG = angular gyrus. amPFC = anterior medial prefrontal cortex. LH = left hemisphere. Functional connectivity units are Fisher z-scores. 95% confidence intervals are displayed. Results from the left hemisphere are shown here for illustrative purposes; complete results from both hemispheres are described in the text.
